# Mapping Community Opioid Exposure Through Wastewater-Based Epidemiology as a Means to Engage Pharmacies in Harm Reduction Efforts

**DOI:** 10.5888/pcd17.200053

**Published:** 2020-08-20

**Authors:** Claire Duvallet, Bryan D. Hayes, Timothy B. Erickson, Peter R. Chai, Mariana Matus

**Affiliations:** 1 Biobot Analytics, Inc, Somerville, Massachusetts; 2 Department of Pharmacy, Massachusetts General Hospital, Boston, Massachusetts; 3 Department of Emergency Medicine, Harvard Medical School, Boston, Massachusetts; 4 Division of Medical Toxicology, Department of Emergency Medicine, Brigham Health, Boston, Massachusetts; 5 Harvard Humanitarian Institute, Boston, Massachusetts; 6 The Fenway Institute, Boston, Massachusetts; 7 The Koch Institute for Integrated Cancer Research, Massachusetts Institute of Technology, Cambridge, Massachusetts

**Figure:**
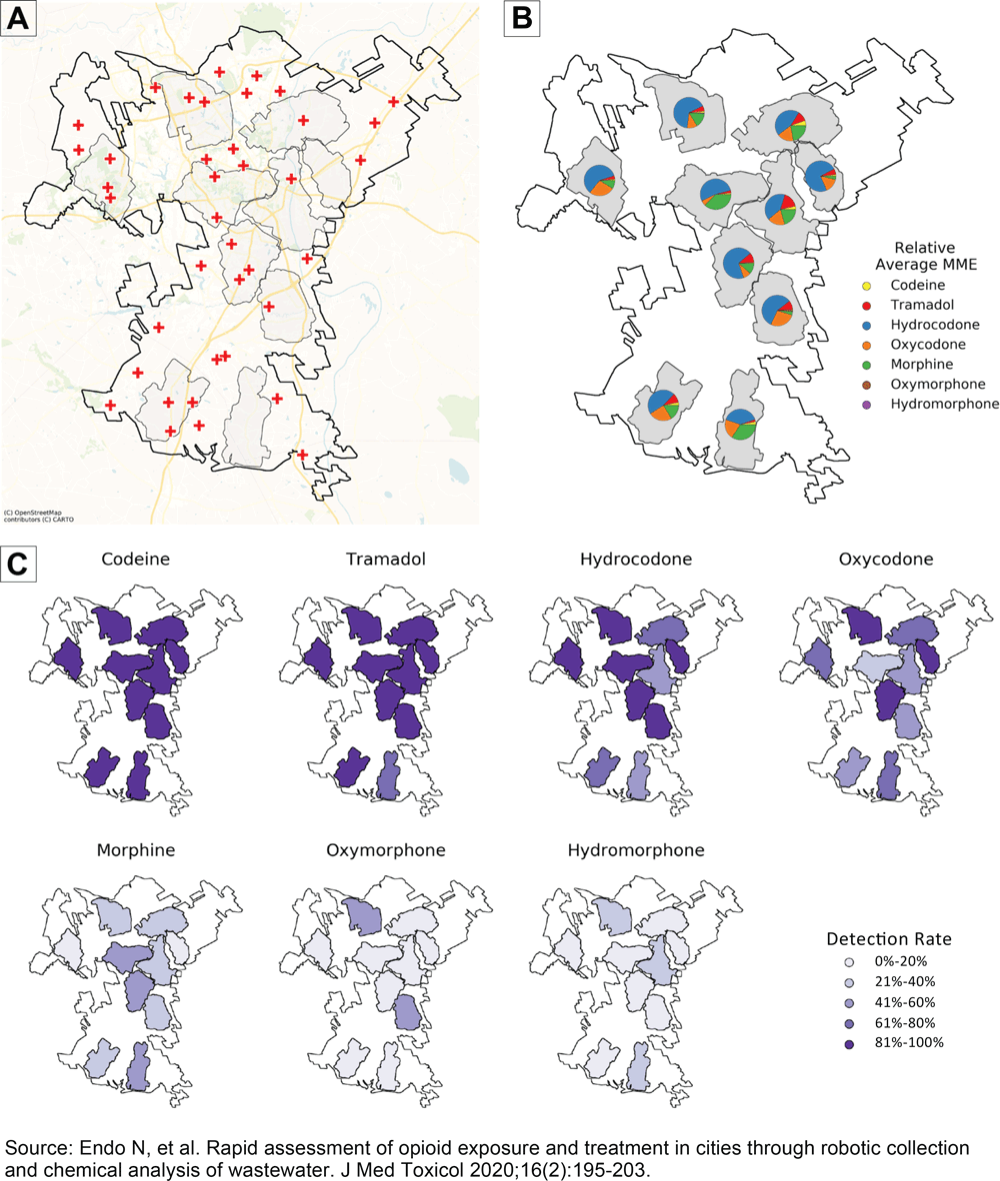
Map. Wastewater-based monitoring of opioid exposure from a pilot study conducted in North Carolina, June–November 2018. Opioid exposure was determined by measuring the concentration of opioid metabolites in sewage using LC-MS/MS. Mapping exposure within cities highlights priority substances and areas for tailoring harm reduction efforts. Map A shows anonymized outline of the municipality, sampling locations, and pharmacies. Map B shows relative average exposure to prescription opioids, highlighting priority substances in each location. Map C shows detection rates for each opioid, showing geographic patterns of opioid use and identifying municipality-wide priorities. All geographical data are anonymized, are for illustrative purposes only, and have no relation to the original location of the study. Abbreviation: LC-MS, liquid chromatography–mass spectrometry; MME, morphine milligram equivalents; MS, mass spectrometry.

## Background

The opioid epidemic is an unprecedented public health crisis in the United States. Community pharmacies are important stakeholders in detecting and addressing opioid misuse. Neighborhood pharmacies are the primary distributors of naloxone and are critical access points for individuals who get opioid prescriptions filled (1). They are ideal locations where referral to substance use disorder treatment, initiation of medication assisted therapy, or community outreach can occur (2,3). Despite this, pharmacies are not fully integrated into opioid response; pharmacists have less information than physicians do about the local milieu of nonmedical opioid use (4–6). Mapping opioid exposure in areas surrounding pharmacies can provide important insights to pharmacists about the prevalence and patterns of opioid use and potential misuse. These maps may also enable pharmacies to function as novel, nontraditional sites that link individuals who have opioid use disorder to formal treatment programs (7).

Visualizing opioid exposure in communities can be accomplished using wastewater-based epidemiology (WBE) (8,9). In WBE, the concentrations of metabolites excreted after drug exposure are measured from city sewers, providing naturally de-identified data on opioid exposure within a community. Combined with geographic information systems techniques, maps of community-level opioid exposure and potential hidden populations of opioid use can be generated. These maps can in turn provide an evidentiary basis for deployment of pharmacy-centered public health responses.

## Data and Methods

The maps depict anonymized results from a pilot study of wastewater opioid monitoring in a municipality in North Carolina from June through November 2018. All geographies were manually distorted at the request of the municipality to preserve anonymity. The map presented has no relation to the original pilot study location. Pharmacy locations represent the same total number and number in each sampling location as in the original study but are otherwise randomly located (Map A). In the pilot study, discussions with the municipality were based on the original non-anonymized maps.

Wastewater data were collected and processed as described by Endo et al (8). Twenty-four-hour aggregate samples were collected from 10 residential manholes every 2 weeks from June through August and monthly from September through November 2018. Opioid metabolites were measured and quantified using LC-MS/MS (liquid chromatography–mass spectrometry/mass spectrometry) and converted to morphine milligram equivalents (MME) as described previously (8,9). Relative average MME (Map B) was calculated by taking the average of each opioid over the sampling period. Detection rates (Map C) were calculated as the number of non-zero samples divided by total successful samples in each location. Maps were created in Python 3 (Python Software Foundation). Code to reproduce these analyses is available at https://github.com/biobotanalytics/gis-snapshot-opioids-public.

## Highlights

Map A provides an overview of the sampling locations and pharmacies within the community. Map B quantifies opioids relative to one another in each sampling location after correcting for potency (ie, by converting to MME) and highlights priority substances in each location so that pharmacists can counsel individuals seeking opioids of potential misuse in their neighborhood. Map C shows detection rates of select opioids, allowing for citywide comparisons. This visualization demonstrates patterns of drug exposure across a community, highlights areas with particularly high or low exposures to different opioids, and indicates which opioids have ubiquitous community-level exposure versus those with geographical specificity.

These maps display 3 points that are immediately actionable by public health officials and pharmacies. First, they highlight key opioids of importance in specific areas within a community. For example, oxycodone exposure varies throughout the city (Maps B and C), which could be a result of differences in community consumption, prescribing practices, or drug availability. Such maps may trigger pharmacies to initiate oxycodone-specific drug “take-back” programs in areas with high exposure to this opioid. Second, WBE maps reflect the pattern of opioid exposure across a city. For example, despite being ubiquitously detected across all sampling locations (Map C), codeine does not contribute much to the overall opioid burden (Map B). This may be in part driven by codeine’s low potency relative to the other opioids (codeine has an MME of 0.15; 6.7 mg of codeine is equivalent to 1 mg of morphine) or by technical factors that result in more reliable detection of its metabolite in wastewater. In contrast, hydrocodone contributes to more than half of the total opioid burden in all sampling locations (Map B), so targeting it rather than codeine may be more effective for interventions like drug take-back campaigns. Third, monitoring and mapping community-level wastewater-based opioid exposure over time can indicate when specific opioids enter the community and inform pharmacists in assessing the effectiveness of their opioid-related outreach and harm reduction efforts.

## Action

Maps of wastewater-based opioid exposure within cities can be used to target new policies and programs geographically within a community, facilitate partnerships and coordination between stakeholders involved in opioid response efforts, monitor the effect of interventions on community health over time, and tailor educational materials to the substances being consumed in each community. In this pilot study, wastewater-based data were used to initiate more than 30 community conversations about opioid use disorder with neighborhood and civic groups, churches, and the chamber of commerce; modify public educational and outreach materials in local and national media outlets; and inform drug disposal locations for National Prescription Drug Take Back Day, leading to a twofold increase in the prescription medications taken back by community leaders (10).

WBE can also be used to integrate pharmacies into the opioid response. For example, WBE could be used to find pharmacies frequented by opioid “shoppers” (11). Pharmacies with many more prescriptions filled than their community’s respective opioid exposure could be flagged as potential “shopping” locations and their opioid prescribing policies subsequently reviewed. Additionally, despite standing orders that authorize most US pharmacies to dispense naloxone, inadequate training and lack of patient education prevents many from doing so (12). Communities with high overdose rates but low wastewater-based naloxone exposure could be promising targets for educational campaigns, supporting pharmacists in those communities to dispense naloxone and educating the public on its availability. 

Finally, hidden populations with opioid use disorder who overdose and receive naloxone but do not present to traditional health care centers may present to pharmacies for naloxone refills. These and other vulnerable populations could benefit from pharmacy-centered interventions informed by WBE maps (13). WBE fills important data gaps, providing information on all individuals within a community regardless of their access to health care, and informs pharmacists about opioid use in their local communities. Together, WBE and mapping techniques provide actionable information for public health officials, pharmacies, and other stakeholders involved in opioid response efforts.
